# Root transcriptional dynamics induced by beneficial rhizobacteria and microbial immune elicitors reveal signatures of adaptation to mutualists

**DOI:** 10.1111/tpj.13741

**Published:** 2017-11-15

**Authors:** Ioannis A. Stringlis, Silvia Proietti, Richard Hickman, Marcel C. Van Verk, Christos Zamioudis, Corné M. J. Pieterse

**Affiliations:** ^1^ Plant‐Microbe Interactions Department of Biology Faculty of Science Utrecht University P.O. Box 800.56 3508 TB Utrecht the Netherlands; ^2^ Present address: Department of Ecological and Biological Sciences University of Tuscia Viterbo Italy; ^3^ Present address: Keygene N.V. P.O. Box 216 6700 AE Wageningen the Netherlands; ^4^ Present address: Rijk Zwaan Breeding B.V. P.O. Box 40 2678 ZG De Lier the Netherlands

**Keywords:** plant growth‐promoting rhizobacteria, root microbiome, time series RNA‐Seq, transcriptomics, *Arabidopsis thaliana*, *Pseudomonas simiae *WCS417, flagellin, chitin, auxin, growth‐defense trade‐off

## Abstract

Below ground, microbe‐associated molecular patterns (MAMPs) of root‐associated microbiota can trigger costly defenses at the expense of plant growth. However, beneficial rhizobacteria, such as *Pseudomonas simiae* WCS417, promote plant growth and induce systemic resistance without being warded off by local root immune responses. To investigate early root responses that facilitate WCS417 to exert its plant‐beneficial functions, we performed time series RNA‐Seq of Arabidopsis roots in response to live WCS417 and compared it with MAMPs flg22^417^ (from WCS417), flg22^Pa^ (from pathogenic *Pseudomonas aeruginosa*) and fungal chitin. The MAMP transcriptional responses differed in timing, but displayed a large overlap in gene identity. MAMP‐upregulated genes are enriched for genes with functions in immunity, while downregulated genes are enriched for genes related to growth and development. Although 74% of the transcriptional changes inflicted by live WCS417 overlapped with the flg22^417^ profile, WCS417 actively suppressed more than half of the MAMP‐triggered transcriptional responses, possibly to allow the establishment of a mutually beneficial interaction with the host root. Interestingly, the sector of the flg22^417^‐repressed transcriptional network that is not affected by WCS417 has a strong auxin signature. Using auxin response mutant *tir1afb2afb3*, we demonstrate a dual role for auxin signaling in finely balancing growth‐promoting and defense‐eliciting activities of beneficial microbes in plant roots.

## Significance Statement

Using time‐series transcriptomics, this work provides novel insight into how beneficial microbes in the rhizosphere suppress growth‐repressing defense responses that are triggered in the roots by immune elicitors of the plethora of soil‐borne microbiota on and in roots. The work pinpoints a dual role for auxin signaling in finely balancing growth‐promoting and defense‐eliciting activities of beneficial microbes in plant roots.

## Introduction

Through photosynthesis, plants have the ability to convert sunlight into chemical energy and provide other organisms with food and oxygen, both above ground and below ground. Below ground, roots interact with an astonishing number of microbiota, also called the rhizosphere microbiome (Berendsen *et al*., 2012; Bulgarelli *et al*., 2013). Via root exudations, plants invest up to 20% of the photosynthetically fixed carbon sources in the preservation of these rhizosphere microbiota (Bais *et al*., 2006; Philippot *et al*., 2013). In return, beneficial rhizosphere microbiota improve root architecture, enhance nutrient uptake and stimulate the plant's immune system (Lugtenberg and Kamilova, 2009; Berendsen *et al*., 2012; Pieterse *et al*., 2016; Venturi and Keel, 2016). Classical examples of such beneficial microbes are mycorrhizal fungi, Rhizobium bacteria, and plant growth‐promoting rhizobacteria (PGPR) and fungi (PGPF; Zamioudis and Pieterse, 2012).

In the past two decades, the interaction between the PGPR *Pseudomonas simiae* WCS417 (WCS417; formerly known as *Pseudomonas fluorescens* WCS417; Berendsen *et al*., 2015) and the model plant *Arabidopsis thaliana* (Arabidopsis) was intensively studied to elucidate the molecular mechanisms underlying plant‐beneficial effects of this PGPR on plant growth and immunity (Pieterse *et al*., 2014). Colonization of Arabidopsis roots by WCS417 promotes plant growth by driving auxin‐dependent developmental changes in root architecture, resulting in the stimulation of lateral root emergence and root hair formation (Zamioudis *et al*., 2013). Colonization of Arabidopsis roots by WCS417 also triggers an induced systemic resistance (ISR) that is effective against a broad spectrum of pathogens (Pieterse *et al*., 2014). WCS417‐ISR is not associated with the direct activation of costly defenses, but with a phenomenon called defense priming (Martinez‐Medina *et al*., 2016). WCS417‐primed plants display an accelerated defense response upon pathogen or insect attack but, in the absence of an invader, costly defenses are not expressed. The ability of PGPRs to simultaneously promote growth and immunity, without the antagonistic features that are typically associated with growth‐defense tradeoffs (Huot *et al*., 2014), make these beneficial soil microbes ideal for the development of biological control agents.

Although generally beneficial for plants, PGPR and PGPF possess microbe‐associated molecular patterns (MAMPs) that, like MAMPs of pathogens, can be recognized by pattern recognition receptors (PRRs) of the plant immune system (Jones and Dangl, 2006; Dodds and Rathjen, 2010). Well‐characterized MAMPs are flagellin, the principle component of bacterial flagella, which is recognized by the PRR FLAGELLIN‐SENSITIVE2 (FLS2; Gomez‐Gomez *et al*., 1999; Boller and Felix, 2009), and the fungal cell wall component chitin, which is recognized by the PRR CHITIN ELICITOR RECEPTOR KINASE1 (CERK1; Miya *et al*., 2007). MAMP recognition by PRRs results in the induction of a coordinated set of defense responses, called MAMP‐triggered immunity (MTI). MTI forms a first line of defense against pathogen invasion (Macho and Zipfel, 2014). Purified flagellin from PGPR strains *P. simiae* WCS417, *Pseudomonas capeferrum* WCS358 and *Pseudomonas defensor* WCS374 (previously known as *Pseudomonas putida* WCS358 and *P. fluorescens* WCS374, respectively; Berendsen *et al*., 2015) elicited typical MTI responses in tobacco suspension cells, such as generation of active oxygen species, extracellular medium alkalization and activation of defense‐related gene expression (Van Loon *et al*., 2008). In Arabidopsis roots, heat‐killed *P. simiae* WCS417 bacteria activated the expression of the MTI marker gene *CYP71A12* and triggered the deposition of callose in exposed root cells to the same extent as did the 22‐amino acid defense elicitor‐active epitope flg22^Pa^ from the opportunistic pathogen *Pseudomonas aeruginosa* PO1 (Millet *et al*., 2010). Similarly, flg22^PsJN^ derived from flagellin of the endophytic PGPR *Burkholderia phytoformans* PsJN induced MTI responses in grapevine and Arabidopsis (Trda *et al*., 2014). Also, MAMPs of the PGPF *Piriformospora indica* activated strong MTI responses in Arabidopsis roots (Jacobs *et al*., 2011), indicating that both PGPR and PGPF in the rhizosphere are potential activators of local root immune responses. As a result of the activation of energy‐costly defense mechanisms, exposure to MAMPs typically has a negative effect on plant growth (Gomez‐Gomez *et al*., 1999; Pel and Pieterse, 2013). Nevertheless, PGPR and PGPF, which abundantly interact with plant roots, promote plant growth rather than suppressing it. Hence, beneficial microbes must have evolved strategies to reduce stimulation of local host immune responses or to actively suppress MTI (Van Wees *et al*., 2008; Zamioudis and Pieterse, 2012; Trda *et al*., 2015).

In this study, we used time series RNA‐Seq to investigate the early transcriptional responses of Arabidopsis roots to living plant growth‐promoting and ISR‐inducing WCS417 bacteria in comparison to its MAMP flg22^417^. We show that the root response to flg22^417^ is highly similar to that of flg22^Pa^ of the pathogen *P. aeruginosa* and that of fungal chitin. Transcriptional changes inflicted by living WCS417 overlap largely with those mediated by the MAMPs, but about half of the MAMP‐induced transcriptional changes are suppressed by living WCS417 cells. The MAMP‐repressed genes that are not affected by WCS417 have a strong auxin signature. We provide evidence that points to a dual role for auxin in finely balancing root growth and defense responses to PGPR.

## Results

### Time series RNA‐Seq reveals kinetics of transcriptional changes to live WCS417 and MAMPs

**Figure 1:**
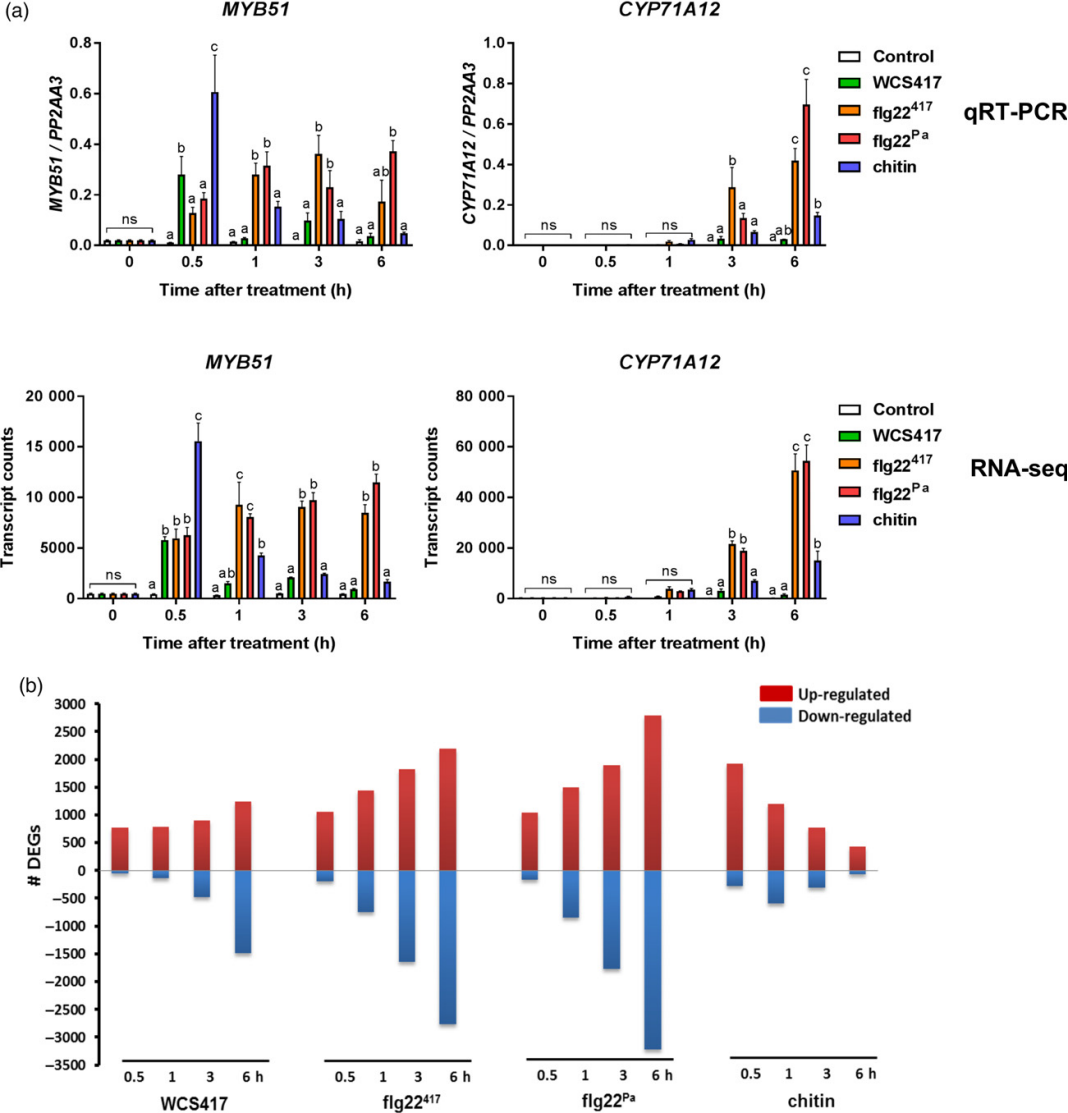
Expression profiles of microbe‐associated molecular pattern (MAMP)‐triggered immunity (MTI) marker genes *MYB51* and *CYP71A12*, and the number of differentially expressed genes (DEGs) in roots in response to WCS417, flg22^417^, flg22^Pa^ and chitin. (a) Gene expression profiles of *MYB51* and *CYP71A12* in WCS417‐, flg22^417^‐, flg22^Pa^‐ and chitin‐treated Arabidopsis Col‐0 roots quantified by quantitative reverse transcriptase‐polymerase chain reaction (qRT‐PCR; top) and RNA‐Seq (bottom). For qRT‐PCR, transcript levels were normalized to that of reference gene *PP2AA3* (At1g13320). Data are means of three biological replicates. Error bars represent SE. Different letters represent statistically significant differences between treatments (two‐way anova, Tukey's test; *P *< 0.05; ns, not significant). (b) Number of induced (red bars) and repressed (blue bars) DEGs from RNA‐Seq analysis of WCS417‐, flg22^417^‐, flg22^Pa^‐ and chitin‐treated Arabidopsis Col‐0 roots at indicated time points after treatment [false discovery rate (FDR) < 0.05; > twofold].

Colonization of Arabidopsis roots by WCS417 bacteria typically stimulates plant growth (Zamioudis *et al*., 2013; Pieterse *et al*., 2014). By contrast, when plant roots are exposed to purified MAMPs, such as flagellin, plant growth is severely inhibited (Gomez‐Gomez *et al*., 1999). We reasoned that a comparison between early transcriptional root responses to live WCS417 and its MAMP flg22^417^ would provide insight into how WCS417 is able to bypass growth‐defense tradeoffs. To this end, we analyzed whole transcriptome changes in roots of 22‐day‐old Arabidopsis Col‐0 plants at 0.5, 1, 3 and 6 h after elicitation with live WCS417 cells or its MAMP flg22^417^. The well‐characterized MAMPs flg22^Pa^ of *P. aeruginosa* (containing five amino acid differences in comparison to flg22^417^; Figure S1) and fungal chitin were included in the study as well. First, we verified the expression pattern of the root MTI marker genes *MYB51* and *CYP71A12* (Denoux *et al*., 2008; Millet *et al*., 2010) in the harvested root material (Figure 1). The MAMPs flg22^417^, flg22^Pa^ and chitin significantly activated both marker genes in the 6‐h time window tested, albeit with different timing and amplitude. At 0.5 h after treatment, live WCS417 cells activated *MYB51* expression significantly compared with control, after which it quickly returned to basal levels. *CYP71A12* transcript levels remained unchanged after WCS417 treatment. Because the MTI marker genes were significantly activated in response to MAMP treatment, we pursued with the RNA‐Seq analysis. Per treatment and time point, three biological replicates were subjected to RNA‐Seq. Each biological replicate consisted of eight roots that were pooled to form one sample. RNA‐Illumina sequencing yielded on average 30 million reads per sample, of which > 90% aligned to the Arabidopsis genome after quality filtering (Van Verk *et al*., 2013; Hickman *et al*., 2017). The expression profiles of *MYB51* and *CYP71A12* quantified by RNA‐Seq were highly similar to those quantified by quantitative reverse transcriptase‐polymerase chain reaction (qRT‐PCR; Figure 1a), confirming that the RNA‐Seq analysis was performed correctly.

### Magnitude and timing of early root transcriptional responses

Differentially expressed genes (DEGs) were selected from each treatment and time point according to their significance in fold change expression [false discovery rate (FDR) < 0.05] and an additional threshold level of at least twofold change (−1 > log_2_ > 1) in comparison to the untreated controls that were harvested at the same time as the treated samples (Dataset S1). RNA‐Seq results revealed that the number of genes that are significantly activated or repressed during the different treatments differs over time (Figure 1b). Within the first 0.5 h, live WCS417 cells predominantly activated the expression of genes. In terms of numbers of DEGs, a similar pattern was observed for flg22^417^ and flg22^Pa^. At later time points, the number of DEGs gradually increased for both the up‐ and down‐regulated genes, although this was notably milder in response to WCS417 (accumulating to a total of 3 559 unique DEGs) than in response to flg22^417^ and flg22^Pa^ (accumulating to a total of 5 934 and 6 955 unique DEGs, respectively). The root response to chitin showed a different pattern. The number of DEGs peaked already at 0.5 h after chitin treatment, after which the number of predominantly upregulated DEGs gradually decreased. With a total number of 3 342 unique DEGs, the response to chitin was milder than that of the flg22 MAMPs.

To investigate to what extent the root transcriptional responses to flg22^Pa^ and chitin in our study relate to previous microarray studies in which the response to these MAMPs were analyzed in Arabidopsis roots (Wan *et al*., 2008; Beck *et al*., 2014) or seedlings (Zipfel *et al*., 2004), we selected DEGs from the 0.5 h time point of our flg22^Pa^ and chitin RNA‐Seq datasets. This time point was selected because it overlapped with that used in the published microarray studies. DEGs whose probe sets were not present on the used microarrays were excluded from the selection (Dataset S2). Venn diagrams in Figure S2 show that of the 357 DEGs responding within 0.5 h to flg22^Pa^ in roots of Arabidopsis accession Landsberg *erecta* (L*er‐*0; Beck *et al*., 2014; Dataset S2), 75% was also present in our set of flg22^Pa^‐responding DEGs. For the 932 DEGs responding within 0.5 h to flg22^Pa^ in whole L*er‐*0 seedlings (Zipfel *et al*., 2004; Dataset S2), the overlap with our set of flg22^Pa^‐responding DEGs in roots was 48%. Of the 878 DEGs responding within 0.5 h in Col‐0 roots to the chitin octamer chitooctaose (Wan *et al*., 2008; Dataset S2), 66% was also present in our set of chitin‐responding DEGs. In this study, we significantly expanded the MAMP‐responsive dataset of roots by generating an information‐rich time series of gene expression profiles that allow analysis of the dynamics of co‐expressed gene clusters in the triggered gene transcriptional networks.

### Nature and timing of core root responses to WCS417, flg22^417^, flg22^Pa^ and chitin

**Figure 2:**
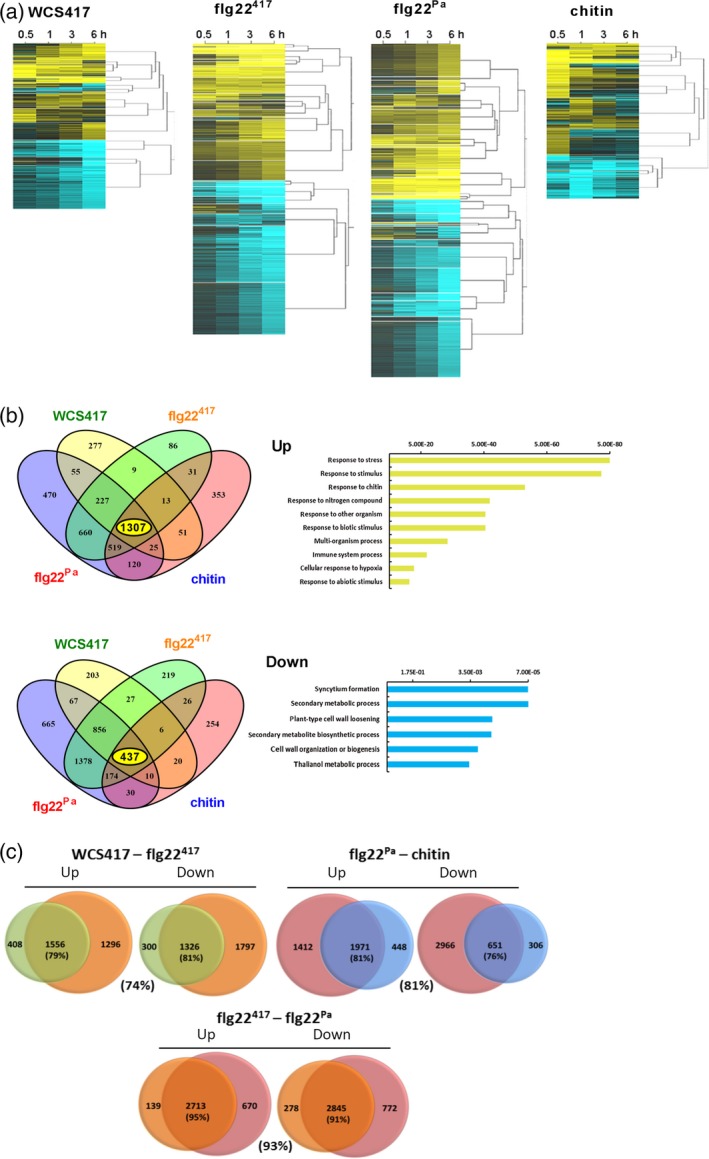
Dynamics and comparative analysis of the root transcriptome in response to live WCS417 cells and the microbe‐associated molecular patterns (MAMPs) flg22^417^, flg22^Pa^ and chitin. (a) Dynamics of the expression patterns of the 3559 WCS417‐, 5934 flg22^417^‐, 6955 flg22^Pa^‐ and 3342 chitin‐responsive differentially expressed genes (DEGs) in Arabidopsis Col‐0 roots. Gene expression is plotted in yellow–blue color scale, with yellow indicating upregulation and blue indicating downregulation in comparison to controls at the same time point. DEGs were clustered using SplineCluster. (b) Venn diagrams showing the pair‐wise overlap between the upregulated and downregulated DEGs of the indicated treatments. Enriched gene ontology (GO) terms associated with the core sets of upregulated (1 307) and downregulated (437) DEGs in all treatments are shown with *P‐*values indicated on *x*‐axes (full data in Dataset S3). (c) Venn diagrams showing the overlap between the upregulated and downregulated genes of the indicated treatments. Percentages indicate the percentage of the smallest group of genes that is shared with the other group of genes in the comparison. The colors of circles in the Venn diagrams are as follows: green = WCS417; orange = flg22^417^; red = flg22^Pa^; blue = chitin.

**Figure 3:**
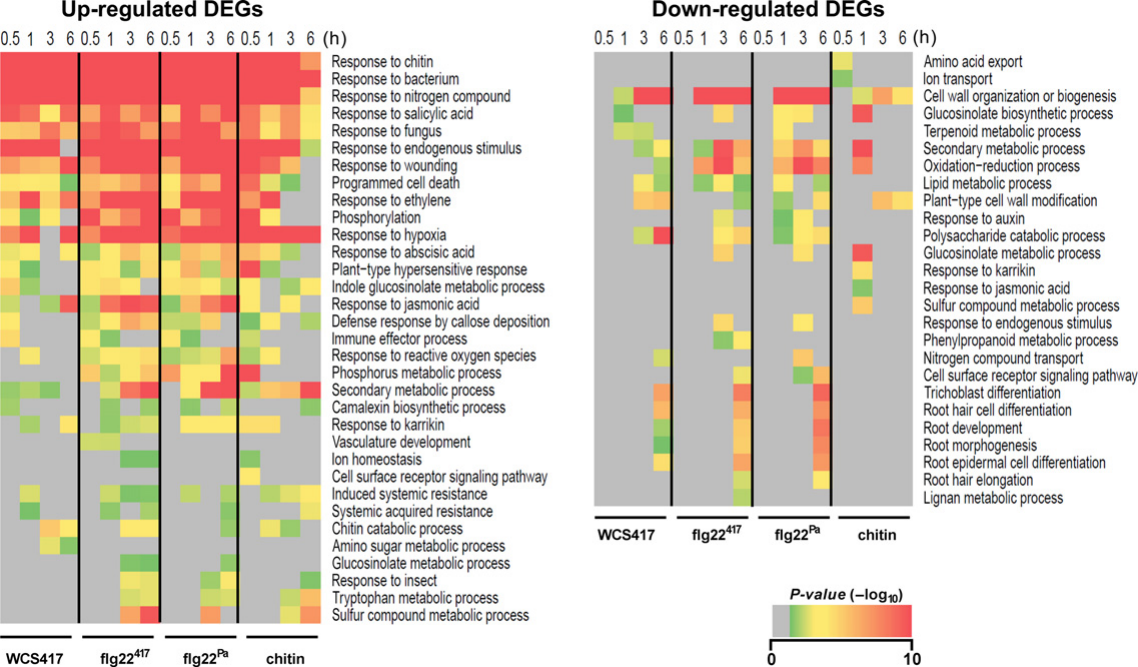
Time course gene ontology (GO) term enrichment analysis of the WCS417, flg22^417^, flg22^Pa^ and chitin differentially expressed genes (DEGs) sets. Heatmaps represent the *P*‐values of GO term overrepresentation of up‐ or down‐regulated DEGs (corresponding to DEGs provided in Dataset S1) in response to treatment of the Arabidopsis roots with WCS417, flg22^417^, flg22^Pa^ or chitin at given time points. On the right, overrepresented GO terms. The significance was plotted in gray–red color scale, with gray indicating no significance and red high significance.

To investigate to what extent genes and biological processes are shared between the four treatments, we compared their DEGs. Figure 2 shows heatmaps of the clustered expression profiles of the DEGs at 0.5, 1, 3 and 6 h after treatment of the roots with WCS417, flg22^417^, flg22^Pa^ or chitin. Figure 2(b) shows the overlap in DEGs in the up‐ and down‐regulated gene sets. Of the union of 4 203 upregulated genes, a core set of 1 307 genes are upregulated; whereas of the union of 4 372 downregulated genes, a core set of 437 are downregulated in response to all four treatments. Among the core sets of shared DEGs are several well‐characterized MAMP‐responsive marker genes, including *CYP71A12* (At2g30750), *FRK1* (At2g19190), *PROPEP2* (At5g64890) and *MPK11* (At1g01560). Gene ontology (GO) analysis (Boyle *et al*., 2004) of overrepresented biological processes in the core set of DEGs shows that the upregulated DEGs are predominantly associated with stress and defense, while the core set of downregulated genes are predominantly related to growth and development (Dataset S3). A similar pattern can be observed in the significance analysis of the GO terms that are enriched in each treatment over time (Figure 3). GO terms related to responses to stress elicitors are clearly most significantly enriched in the upregulated set of DEGs, while GO terms related to cell wall biogenesis and root development are most prominently enriched in the downregulated set of DEGs.

### WCS417 versus flg22^417^

In a more detailed analysis, we performed pair‐wise comparisons between the sets of up‐ and down‐regulated DEGs of the four treatments. Figure 2(c) shows that there is a large overlap between the DEGs of the WCS417 and flg22^417^ treatment. Of the 1964 WCS417‐induced genes, 79% are also activated in response to flg22^417^ treatment. For the 1626 downregulated genes, this percentage is similarly high (81%). When comparing the significance of the enriched GO terms in the up‐ and down‐regulated sets of DEGs of both treatments over time (Figure 3), the nature of the root response to live WCS417 cells and flg22^417^ is rather similar. However, while many of the GO terms in the upregulated set of DEGs remain significantly overrepresented in the flg22^417^‐treated roots over time, they disappear at later time points in the WCS417‐induced set of DEGs. Interestingly, a large proportion of the 5934 DEGs responding to flg22^417^ treatment (50%) were not responding to WCS417 treatment, suggesting that the flg22^417^‐responsiveness of these DEGs is suppressed by live WCS417 bacteria. The nature of the differential root response to live WCS417 cells and flg22^417^ is analyzed in more detail below.

### flg22^417^ versus flg22^Pa^

The sequence of the 22‐amino acid flg22 peptides of the mutualist *P. simiae* WCS417 and the pathogen *P. aeruginosa* PAO1 differs at five of the 22 positions (Figure S1). Previous studies have shown that replacement of specific amino acids in the flg22 sequence of *P. aeruginosa* or other bacterial phytopathogens (black and red arrows, Figure S1) can dramatically affect immune elicitation in tomato and Arabidopsis, and growth inhibition in the latter (Felix *et al*., 1999; Sun *et al*., 2006; Naito *et al*., 2008). One of these amino acids (red arrow, Figure S1) was different in the sequence of flg22^417^ compared with flg22^Pa^. Nevertheless, the set of DEGs in Arabidopsis roots responding to flg22^417^ and flg22^Pa^ show a very high overall overlap, both in numbers and in gene identity (Figure 2c; 95% overlap in the upregulated DEGs, 91% overlap in the downregulated DEGs, 93% overall overlap). When comparing the significance of the enriched GO terms in the up‐ and down‐regulated sets of DEGs of both treatments over time (Figure 3), it is clear that the nature as well as the timing of the root response to flg22^417^ and flg22^Pa^ is highly similar.

### flg22^Pa^ versus chitin

A plethora of soil microbiota possess either flagellin or chitin. Hence, plant roots must be abundantly exposed to both MAMPs. Pair‐wise comparison of the genes that are significantly affected in response to exposure of Arabidopsis roots to flg22^Pa^ or chitin revealed that, although the overall number of DEGs in response to the chitin treatment is considerably lower (3 342 DEGs for chitin versus 6 955 DEGs for flg22^Pa^), a substantial part of the chitin‐responsive genes is also responsive to flg22^Pa^ (Figure 2c; 81% in the upregulated DEGs, 76% in the downregulated DEGs, 81% overall). Comparison of the enriched GO terms in the sets of DEGs of both treatments over time (Figure 3) shows that the nature of the upregulated responses is very similar, although in general they seem to dampen off over time in the chitin‐treated roots. The number of downregulated genes in the chitin set of DEGs is much lower than in the flg22^Pa^ set of DEGs (987 versus 3 647). The GO term analysis (Figure 3) suggests that processes related to root development are downregulated by flg22^Pa^, while they remain unaffected in response to chitin.

### Suppression of MAMP responses by live WCS417 cells

**Figure 4:**
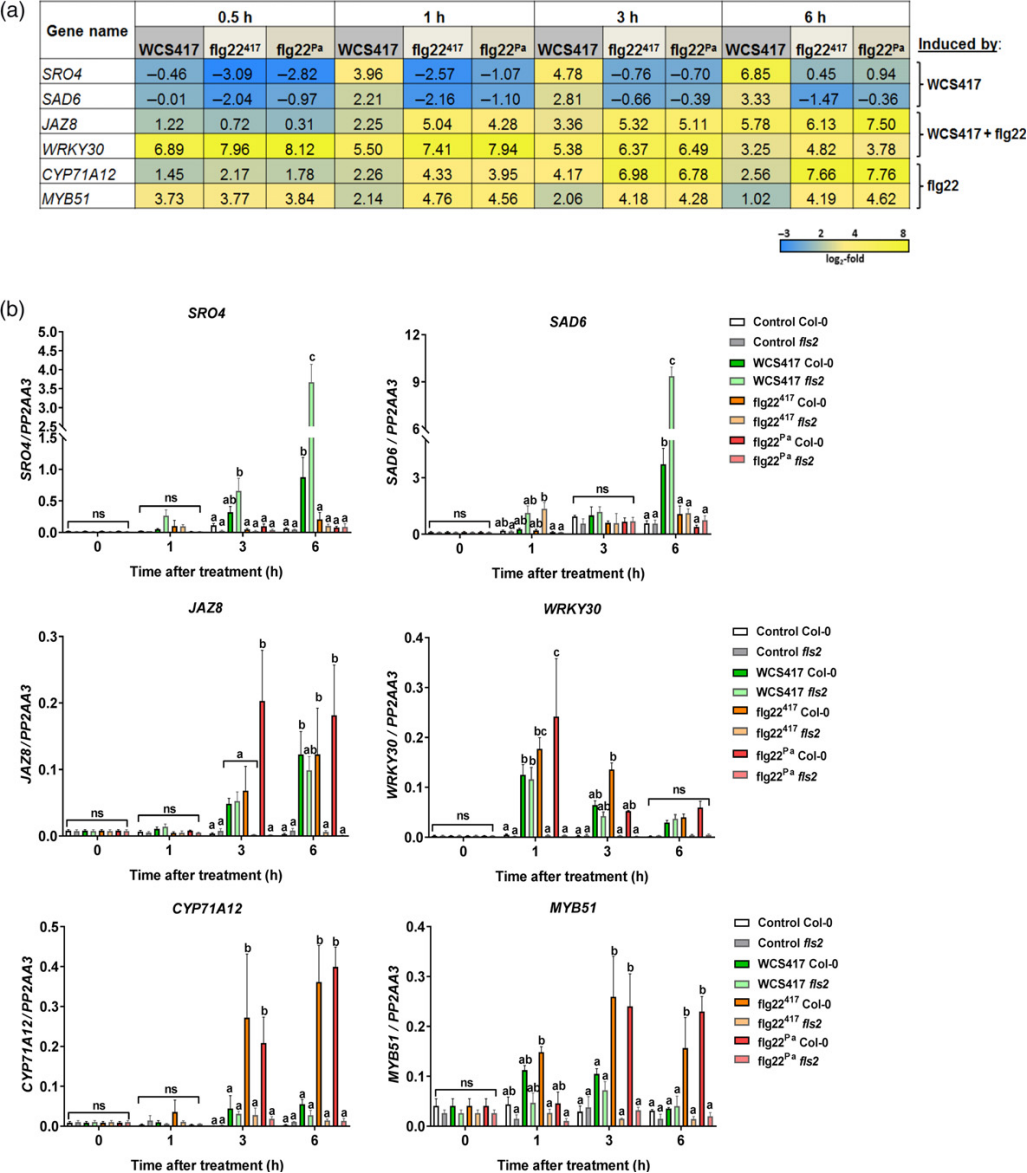
Expression profile of selected genes responsive to WCS417, flg22^417^ and flg22^Pa^ treatments. (a) Expression profile of *SRO4*,*SAD6*,*JAZ8*,*WRKY30*,*CYP71A12* and *MYB51* in Col‐0 roots by RNA‐Seq analysis. Heatmap showing the expression profile in Col‐0 roots at indicated time points after treatment with live WCS417 cells, flg22^417^ or flg22^Pa^ as obtained by RNA‐Seq analysis. Gene expression was plotted in blue–yellow color scale, with blue indicating downregulation or low expression, and yellow indicating high expression. Gene expression was calculated relative to mock (log_2_‐fold change). (b) Expression profile of *SRO4*,*SAD6*,*JAZ8*,*WRKY30*,*CYP71A12* and *MYB51* in Col‐0 and *fls2* roots by quantitative reverse transcriptase‐polymerase chain reaction (qRT‐PCR). Graphs show gene expression levels in Col‐0 or *fls2* roots at indicated time points after treatment with live WCS417, flg22^417^ or flg22^Pa^, as quantified by qRT‐PCR. Gene expression was normalized to the expression level of reference gene *At1g13320*, using the 2^−ΔCt^ method. The shown data are means of three replicates. Error bars represent SE. Different letters represent statistically significant differences between treatments (two‐way anova, Tukey's test; *P *< 0.05; ns, not significant).

Figure 2 shows that the genes that become up‐ or down‐regulated in roots responding to live WCS417 cells are overall similarly responsive to its MAMP flg22^417^, suggesting that the root response to colonization by WCS417 is largely MAMP‐mediated. Besides flagellin, WCS417 bacteria produce more MAMPs, including elongation factor Tu (Kunze *et al*., 2004) and lipopolysaccharides (Zeidler *et al*., 2004), which are likely to elicit partly similar MAMP responses in the roots to flagellin. To investigate whether the observed WCS417 root transcriptional response is flagellin‐specific or merely mediated by the concerted action of multiple WCS417 MAMPs, we selected two genes (*WRKY30* and *JAZ8*) from our RNA‐Seq dataset that were similarly responsive to WCS417 and the flg22 peptides flg22^417^ and flg22^Pa^, two WCS417‐specific genes (*SRO4* and *SAD6*) that were induced by WCS417 but not by the flg22 peptides, and two MAMP‐responsive genes (*MYB51* and *CYP71A12*) that were strongly induced by the flg22 peptides, but are suppressed by live WCS417 cells (Figures 1a and 4). To test whether the expression patterns of the selected genes were flagellin‐specific or not, we monitored their expression pattern in roots of wild‐type Col‐0 and flagellin receptor mutant *fls2* over time by qRT‐PCR. Figure 4(b) shows that the WCS417‐specific genes *SRO4* and *SAD6* were induced by WCS417 in both Col‐0 and *fls2* mutant but not by flg22^417^ and flg22^Pa^, confirming that the roots responded to WCS417. As expected, the flg22‐specific genes *MYB51* and *CYP71A12* followed a FLS2‐dependent expression pattern and were not induced by live WCS417 cells. The WCS417‐ and flg22‐responsive genes *WRKY30* and *JAZ8* were induced in Col‐0 roots by WCS417 and both flg22 peptides. WCS417 induced these genes also in the *fls2* mutant, while the flg22 peptides did not, indicating that these MAMP‐responsive genes can also be induced by other WCS417 MAMPs. These results highlight that WCS417‐mediated root transcriptional responses are not flagellin‐specific but are likely caused by the concerted action of multiple WCS417 MAMPs.

**Figure 5:**
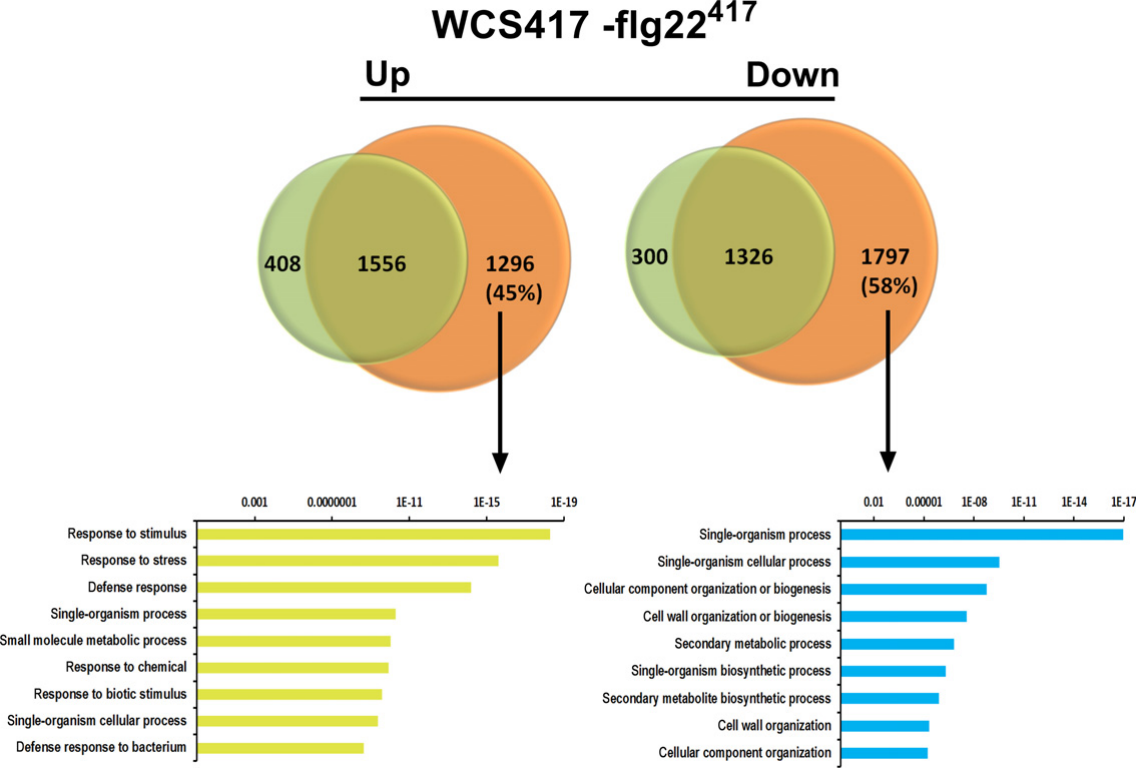
Comparative analysis of the root transcriptome to live WCS417 and flg22^417^. Venn diagrams showing the overlap between the genes upregulated and downregulated upon treatment with WCS417 and flg22^417^. The bottom section shows the most highly enriched gene ontology (GO) terms associated with the sets of differentially expressed genes (DEGs) that are specifically up‐ or downregulated after root exposure to flg22^417^, but not upon colonization of the roots by live WCS417 bacteria. *P*‐values are indicated on the *x*‐axes.

**Figure 6:**
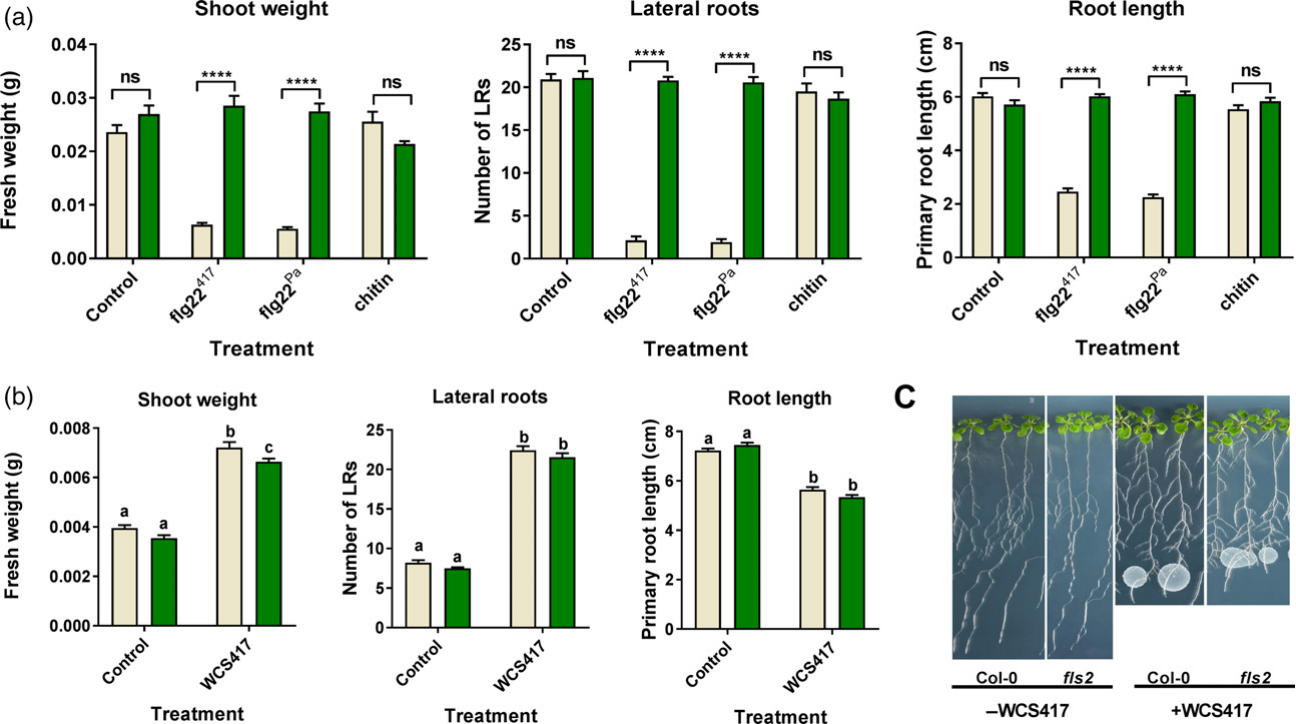
Effect of WCS417, flg22^417^, flg22^Pa^ and chitin on shoot and root growth in Col‐0 and the *fls2* mutant. (a) Effect of flg22^417^, flg22^Pa^ and chitin on shoot and root growth of Col‐0 and *fls2* seedlings. Seven‐day‐old Arabidopsis seedlings were cultivated for 7 days in liquid Murashige and Skoog (MS) medium with 0.5% sucrose, supplemented with or without 500 nm flg22^417^ or flg22^Pa^, or 500 μg ml ^−1^ chitin, after which shoot fresh weight, number of lateral roots formed and primary root length were measured. (b and c) Effect of live WCS417 bacteria on shoot and root growth of Col‐0 and *fls2*. Three‐day‐old Arabidopsis seedlings were cultivated for 7 days on agar‐solidified MS medium with 0.5% sucrose, and inoculated with a 10 μl suspension containing 2 × 10^6^ colony‐forming units (CFU) of WCS417 cells or not inoculated (Control) right below the root tip of each seedling, resulting in rapid colonization of the whole root system. Error bars indicate SE (*n *= 24). Different letters indicate statistically significant differences between Col‐0 and *fls2* after control and WCS417 treatment (two‐way anova, Tukey's test; *P *< 0.05), and asterisks indicate significant difference between Col‐0 and *fls2* plants treated with the same elicitor (two‐way anova, Sidak's test; *^***^ *P *< 0.0001; ns, not significant).

Although the root transcriptional response to live WCS417 bacteria largely overlaps with the root response to its MAMP flg22^417^, more than 50% of the 5 934 flg22^417^‐responsive genes are not responsive to WCS417, suggesting that live cells actively suppress a significant part of the MAMP response. GO term analysis of the set of flg22^417^‐responsive genes that is not responsive to WCS417 (Figure 5) is predominantly associated with defense, suggesting that live WCS417 cells actively suppress a significant portion of the defense‐related genes that is potentially activated by its MAMPs. Interestingly, the flg22^417^‐downregulated set of genes that is not downregulated by live WCS417 cells represents GO terms related to cell wall organization, plant growth and development (Figure 5), suggesting that live WCS417 cells actively prevent the MAMP‐mediated suppression of genes that are associated with plant growth. Indeed, treatment of Arabidopsis Col‐0 seedlings with flg22^417^ or flg22^Pa^ strongly reduced shoot fresh weight, lateral root formation and root length (Figure 6). Mutant *fls2* did not display these flg22‐mediated growth retardations, confirming that this is a flg22‐mediated response. Chitin, which in contrast to flg22^417^ and flg22^Pa^ did not downregulate GO terms related to growth and development (Figure 5), also did not reduce shoot fresh weight, lateral root formation and root length (Figure 6a). Interestingly, in both Col‐0 and *fls2*, treatment of the roots with live WCS417 cells increased shoot fresh weight and the number of lateral roots formed, while mildly reducing primary root length (Figure 6b and c). These results suggest that the suppression of the flg22^417^‐mediated transcriptional response by live WCS417 cells is partially directed towards bypassing the plant growth‐repressing effects of MAMP recognition.

### Auxin signature in early root response to WCS417

**Figure 7:**
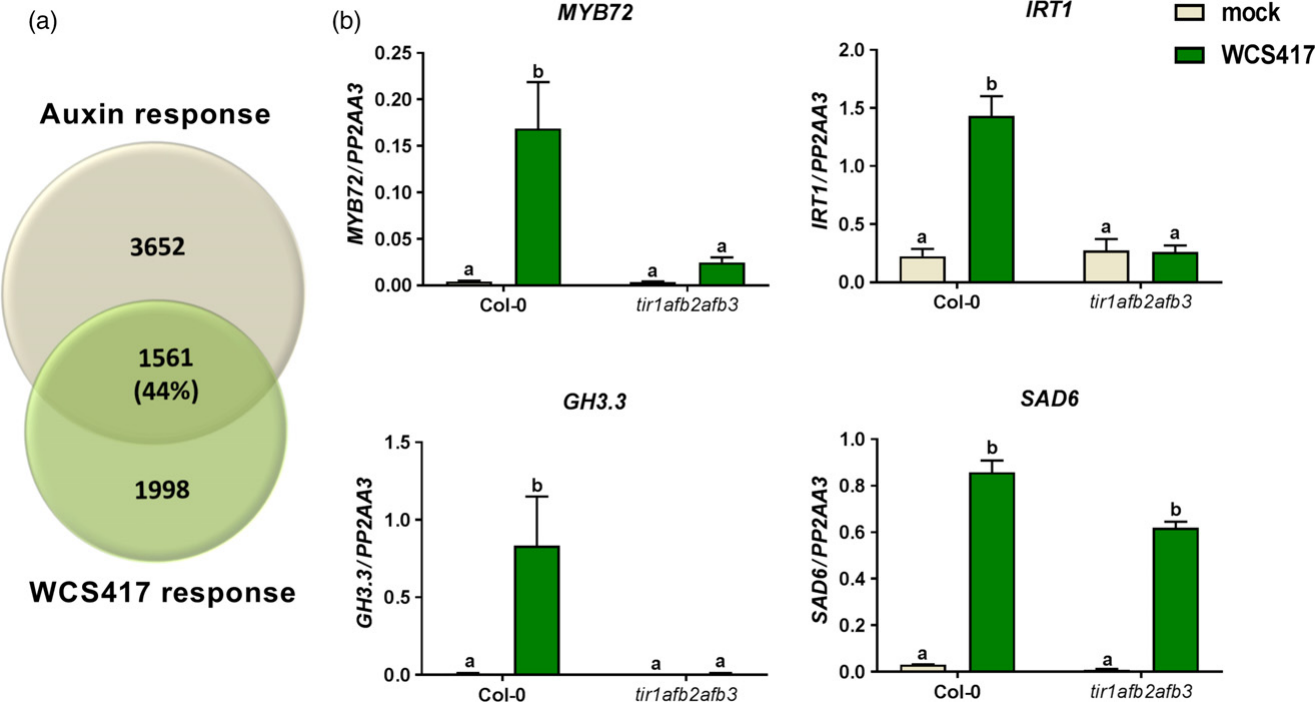
Auxin signatures in the early transcriptome of WCS417‐colonized roots and auxin signaling‐dependent induction of the induced systemic resistance (ISR) markers *MYB72* and *IRT1*. (a) Venn diagram showing the overlap between genes responding to indole‐3‐acetic acid (IAA; auxin) and WCS417 in Arabidopsis roots. The effect of auxin was tested 4 h after treatment of the roots with 5 μm IAA (dataset from Chaiwanon and Wang, 2015), while the effect of WCS417 was tested at all time points after application of the rhizobacteria. (b) Expression levels of *MYB72, IRT1, GH3.3* and *SAD6* as quantified by quantitative reverse transcriptase‐polymerase chain reaction (qRT‐PCR). Expression was tested in roots of 14‐day‐old Col‐0 and *tir1afb2afb3* seedlings at 48 h after inoculation with WCS417. Gene expression levels were normalized to that of reference gene *At1g13320* using the 2^−ΔCt^ method. The shown data are means of three biological replicates. Error bars represent SE. Different letters represent statistically significant differences between treatments (two‐way anova, Tukey's test; *P *< 0.05).

The positive effects on plant growth and root architecture mediated by colonization of the roots by WCS417 (Figure 6b and c) were previously shown to be regulated by auxin, as WCS417 colonization resulted in the activation of the *DR5::YFP* reporter gene in roots, and the auxin response triple mutant *tir1afb2afb3* did not display these growth responses upon treatment with WCS417 (Zamioudis *et al*., 2013). Also, the GO term analysis of the set of DEGs that was suppressed by flg22^417^ but not by live WCS417 cells yielded a number of significantly enriched GO terms related to growth and development, while this gene set contained a considerable number of genes involved in auxin‐related processes (Figure 5; Dataset S4). To investigate whether this role of auxin in WCS417‐mediated root responses was also apparent in the WCS417‐triggered root transcriptome, we compared the WCS417‐responsive set of DEGs (all four time points) with genes from the study of Chaiwanon and Wang (2015), in which transcriptome changes were analyzed in Arabidopsis roots 4 h after treatment with the auxin analog indole‐3‐acetic acid (IAA; Figure 7; Dataset S5). This comparison revealed that 44% of the WCS417‐responsive DEGs (1 561 genes) are also responsive to auxin. GO term analysis of the shared set of DEGs showed that this gene set is associated with cell organization, growth and secondary metabolism (Dataset S5). This finding confirms that the early root response to live WCS417 bacteria has a strong auxin signature, which may contribute to the growth‐promoting effect of WCS417.

Besides promoting plant growth, WCS417 is also a potent elicitor of ISR. Recently, it was demonstrated that the onset of ISR in Arabidopsis and tomato coincides with the activation of the iron deficiency response in colonized roots (Zamioudis *et al*., 2015; Martinez‐Medina *et al*., 2017). The onset of both ISR and the iron deficiency response by ISR‐inducing rhizobacteria is marked by the activation of the root‐specific transcription factor gene *MYB72* and the high‐affinity iron transporter gene *IRON‐REGULATED TRANSPORTER1* (*IRT1*; Van der Ent *et al*., 2008; Zamioudis *et al*., 2015). Because previously auxin has been shown to be essential for the activation of the iron deficiency response in Arabidopsis roots (Chen *et al*., 2010), we reasoned that the WCS417‐induced auxin response in Arabidopsis roots observed in this study might be at the basis of the activation of the iron deficiency response that is required for the onset of ISR. To test this, we assessed the expression of *MYB72* and *IRT1* in roots of wild‐type Col‐0 and the auxin response triple mutant *tir1afb2afb3*, 48 h after treatment of the roots with WCS417. As controls we also tested the expression of the well‐characterized auxin‐responsive gene *GH3.3*, and the auxin‐independent WCS417‐responsive gene *SAD6* (Figure 7b). As expected, the auxin‐independent gene *SAD6* was induced by WCS417 in both Col‐0 and the triple mutant, indicating that the WCS417 treatment worked correctly. Furthermore, the ISR and iron deficiency response marker genes *MYB72* and *IRT1*, and the auxin‐responsive marker gene *GH3.3* were significantly upregulated in Col‐0 roots upon colonization by WCS417. By contrast, their expression was not affected by WCS417 in the roots of the auxin response mutant *tir1afb2afb3* (Figure 7b). Together, these findings suggest an important role for auxin signaling in early root responses to beneficial WCS417 rhizobacteria that are associated with the promotion of plant growth and the onset of ISR.

## Discussion

Roots are essential plant organs responsible for the uptake of nutrients and water, stable anchorage in the soil, and support of shoot growth. Mutualistic and pathogenic interactions of roots with microbiota members in the rhizosphere strongly influence both plant growth and immunity (Berendsen *et al*., 2012). To safeguard their fitness, plant roots need to effectively ward off pathogens and at the same time exploit the profitable functions that are provided by beneficial microbes. Plant roots are continuously exposed to an astonishing number and complexity of microbes, most of which possess MAMPs that potentially activate growth‐retarding immune responses in plant roots (Hacquard *et al*., 2017). Yet, in general plants profit from their root‐associated microbiota by simultaneously displaying enhanced growth and primed systemic immunity. To investigate the mechanisms behind this phenomenon, we compared the early root response of Arabidopsis to colonization by the PGPR and ISR‐eliciting rhizobacterium *P. simiae* WCS417 with those of the purified MAMP elicitors flg22^417^, flg22^Pa^ and chitin. Using time series RNA‐Seq, we monitored the dynamics of the Arabidopsis root response to live WCS417 cells and the three MAMPs, yielding gene expression profiles that markedly expand those previously obtained that only analyzed one or two time points (Verhagen *et al*., 2004; Pozo *et al*., 2008; Wan *et al*., 2008; Beck *et al*., 2014). Intriguingly, the transcriptional root response to flg22^417^ of beneficial WCS417 rhizobacteria was essentially similar to flg22^Pa^ of the bacterial pathogen *P. aeruginosa* PAO1 (93% overlap; Figure 2), even though the 22‐amino acid sequence of the flg22 peptides differed at five positions (Figure S1). The root response to fungal chitin was markedly milder, although the overlap with flg22‐triggered transcriptional changes in both gene identity (81% overlap; Figure 2) and GO term enrichment (Figure 3) was very high. This observation is consistent with previous findings in leaves and roots where different MAMPs/pathogens could activate common components of defense signaling pathway and/or affect a significant proportion of the transcriptome in a similar manner (Asai *et al*., 2002; Zipfel *et al*., 2006; Denoux *et al*., 2008; Wan *et al*., 2008; Millet *et al*., 2010).

When comparing the WCS417‐responsive set of DEGs with that of its flagellin epitope flg22^417^ (Figure 2), we discovered that 74% of the 3 559 genes that significantly respond in the roots to WCS417 also respond to flg22^417^. This indicates that the root response to this beneficial rhizobacterium is largely mediated by flagellin or one or more of its other MAMPs, which are likely to activate largely overlapping gene sets in a redundant manner (Figure 4). On the other hand, half of the 5 934 genes that significantly respond to flg22^417^ were not responsive to live WCS417 cells, suggesting that WCS417 actively suppresses the initiation of a large portion of transcriptional changes that are inflicted by its MAMPs. The set of flg22^417^‐upregulated genes that is suppressed by live WCS417 cells represents GO terms that are predominantly associated with defense (Figure 5), indicating that WCS417 actively suppresses the activation of local immune responses that are potentially activated by its MAMPs. Previously, Millet *et al*. (2010) demonstrated that inoculation of Arabidopsis roots with WCS417 prior to treatment of the roots with flg22^Pa^ prevented the activation of the flg22^Pa^‐responsive genes *MYB51* and *CYP71A12*, supporting the notion that beneficial WCS417 bacteria are able to prevent the activation of MAMP‐responsive genes. Also, in our study, both *MYB51* and *CYP71A12* were strongly activated by flg22^417^ and flg22^Pa^ (Figure 1), but in response to WCS417 they were either not induced at all (*CYP71A12*), or only activated at 0.5 h after colonization and rapidly downregulated afterwards to basal levels (*MYB51*). Because MAMP‐induced responses can result in the inhibition of plant growth (Figure 6; Gomez‐Gomez *et al*., 1999; Pel and Pieterse, 2013), significantly tempering of this response by WCS417 may minimize the growth‐retarding effect of MAMP recognition by plant roots. Moreover, suppression of local MTI responses may facilitate colonization of root tissues by the beneficial microbes that suppress it, as was demonstrated for the beneficial fungus *P. indica* (Jacobs *et al*., 2011).

The set of flg22^417^‐downregulated genes that are not downregulated by WCS417 largely represents GO terms associated with plant growth and development (Figure 5). Hence, downregulation of this sector of the flg22‐responsive transcriptome may reflect the growth‐inhibiting effect of flg22‐recognition on Arabidopsis seedlings (Figure 6; Gomez‐Gomez *et al*., 1999; Pel and Pieterse, 2013; Beck *et al*., 2014). By preventing MAMP‐mediated suppression of this sector of the root transcriptome, live WCS417 cells may further contribute to the promotion of plant growth. This is supported by the observation that Arabidopsis seedlings of which the roots were treated with flg22^417^ or flg22^Pa^ showed a strong FLS2‐dependent growth reduction, while treatment of the roots with live WCS417 cells promoted plant growth in an FLS2‐independent manner (Figure 6).

Of the 3559 WCS417‐responsive genes, 44% were also identified as responsive to auxin in the roots of Arabidopsis (Figure 7a; Chaiwanon and Wang, 2015). This auxin signature in the WCS417‐triggered root transcriptome corroborates with previous findings that showed that WCS417 induced the auxin‐responsive reporter *DR5::YFP* in Arabidopsis roots and that the WCS417‐mediated plant growth‐promoting effect was blocked in the auxin response mutant *tir1afb2afb3* (Zamioudis *et al*., 2013). Together, these findings demonstrate that auxin‐dependent responses in the roots are key to the growth‐promoting effect of WCS417. We further tested whether auxin signaling may also contribute to the onset of early root responses that have been shown to be essential for the development of broad‐spectrum ISR. The onset of WCS417‐induced ISR is associated with by the activation of the ISR marker gene *MYB72* and the iron uptake marker gene *IRT1* in the roots (Van der Ent *et al*., 2008; Zamioudis *et al*., 2015). We showed that activation of both genes by WCS417 is blocked in the triple mutant *tir1afb2afb3* (Figure 7), suggesting that auxin signaling is indeed important in the early stages of ISR initiation in the Arabidopsis root.

In sum, our analysis of the early root transcriptional changes inflicted by live beneficial WCS417 bacteria and purified MAMPs, showed that: (i) the early root response to live WCS417 bacteria is largely driven by its MAMPs; (ii) WCS417 actively suppresses about half of the MAMP‐triggered transcriptional changes; (iii) the root response to the flagellin epitopes flg22^417^ of beneficial WCS417 rhizobacteria and flg22^Pa^ of pathogenic *P. aeruginosa* PAO1 bacteria (differing in five amino acids) are essentially similar; and (iv) auxin plays a dual role in finely balancing the growth‐promoting and systemic immunity‐eliciting activity of this beneficial rhizobacterium. Evasion of MAMP‐responsive transcriptional changes emerged as a major component of the early root response of Arabidopsis to colonization by beneficial WCS417 bacteria. Hence, future research will be directed towards elucidating the bacterial determinant(s) that are responsible for this phenomenon. Detailed knowledge on how beneficial members of the root microbiota simultaneously promote plant growth and immunity will be essential to ultimately utilize their properties in sustainable strategies for crop improvement and crop protection.

## Experimental procedures

### Plant material and growth conditions

*Arabidopsis thaliana* accession Col‐0 and the mutants *fls2* and *tir1afb2afb3* (in Col‐0 background) were used in this study. For all experiments, seeds were surface sterilized (Van Wees *et al*., 2013) and sown on agar‐solidified 1 × Murashige and Skoog (MS) medium supplemented with 0.5% sucrose (Murashige and Skoog, 1962). After 2 days of stratification at 4°C, the Petri dishes were positioned vertically and transferred to a growth chamber (22°C; 10 h light, 14 h dark; light intensity 100 μmol m^−2^ s^−1^).

For testing effects of live WCS417 cells and the MAMPs flg22^417^, flg22^Pa^ and chitin on gene expression, uniform 12‐day‐old seedlings were transferred from MS agar plates to six‐well plates (ø 35 mm per well) containing liquid 1 × MS with 0.5% sucrose, after which they were cultured for 10 more days under the same growth conditions. The day before treatment with WCS417 or MAMP elicitors, the medium of each well was replaced with fresh 1 × MS medium with 0.5% sucrose. Plants were treated when 22 days old. At this age, the plants possessed a well‐developed root system that reproducibly responded to the elicitors and yielded sufficient RNA for the RNA‐Seq gene expression analysis.

For testing the effect of live WCS417 cells and the MAMPs flg22^417^, flg22^Pa^ and chitin on plant growth parameters, uniform 7‐day‐old seedlings were transferred from MS agar plates to six‐well plates containing 1 × MS with 0.5% sucrose (in case of MAMP treatment), or to a fresh 1 × MS agar plate with 0.5% sucrose (in case of treatment with live WCS417 cells). Seedlings were treated when 9 days old. Effects on growth parameters were measured 7 days later.

For testing the role of auxin signaling in WCS417‐mediated expression of ISR marker genes locally in the roots, uniform 10‐day‐old seedlings were transferred from MS plates to square plates (120 × 120 × 17 mm) containing modified agar‐solidified Hoagland medium containing 5 mm KNO_3_, 2 mm MgSO_4_, 2 mm Ca(NO_3_)_2_, 2.5 mm KH_2_PO_4_, 70 μm H_3_BO_3_, 14 μm MnCl_2_, 1 μm ZnSO_4_, 0.5 μm CuSO_4_, 10 μm NaCl, 0.2 μm Na_2_MoO_4_, 4.7 mm MES, 43 mm sucrose and 50 μm Fe‐EDTA (Rodriguez‐Celma *et al*., 2013). The pH was adjusted to 5.5. Seedlings were cultivated for 2 days before treatment with WCS417.

### Cultivation of *Pseudomonas simiae* WCS417 and treatment

*Pseudomonas simiae* WCS417 was cultured at 28°C on King's medium B agar plates (King *et al*., 1954) supplemented with 50 μg ml^−1^ of rifampicin. After 24 h of growth, cells were collected in 10 mm MgSO_4_, washed twice with 10 mm MgSO_4_ by centrifugation for 5 min at 5000 ***g***, and finally resuspended in 10 mm MgSO_4_. For RNA‐Seq and qRT‐PCR gene expression analysis, bacteria were added in each well to a final optical density of 0.1 at 600 nm [OD_600_; 10^8^ colony‐forming units (CFU) ml^−1^]. For measurements of the effect of WCS417 on root and shoot growth, 9‐day‐old Col‐0 and *fls2* seedlings growing on MS agar plates were inoculated by placing a 10 μl suspension of WCS417 at an OD_600_ of 0.002 (2 × 10^6^ CFU ml^−1^) right below the root tip of each seedling. For studying gene expression in roots of Col‐0 and *tir1afb2afb3* seedlings growing on Hoagland agar medium, a 10 μl bacterial suspension at OD_600_ = 0.1 was added below the hypocotyl of each seedling.

### Chemical treatments

For RNA‐Seq and qRT‐PCR analysis of gene expression in Arabidopsis roots in response to MAMPs, defense elicitors were added to the liquid growth medium at the following final concentrations: 1 μm for flg22^417^ and flg22^Pa^, and 1 mg ml^−1^ for chitin (Sigma‐Aldrich, St Louis, MO, USA). For the analysis of the effects of MAMPs on plant growth, the following final concentrations were used: 500 nm for flg22^417^ and flg22^Pa^, and 500 μg ml^−1^ for chitin. The flg22 peptides were added to the liquid MS medium from a 100 μm stock in water. Chitin was added to the MS medium from a 10 mg ml^−1^ stock that was prepared as described (Millet *et al*., 2010). Control plants remained untreated. The amino acid sequence of flg22^417^ was derived from the flagellin‐encoding gene ‘flagellin’ (PS417_19850 – WP_029529076.1) that was extracted from the whole‐genome sequence of WCS417 (Berendsen *et al*., 2015). The flg22^417^ and flg22^Pa^ peptides were synthesized by Proteogenix (Schiltigheim, France) and GenScript (Piscataway, NJ, USA), respectively (purity > 95%).

### Measurement of plant biomass and parameters of root architecture

Col‐0 and *fls2* seedlings were germinated on 1 × MS agar plates with 0.5% sucrose. Seven‐day‐old seedlings were transferred to liquid MS medium with 0.5% sucrose, with or without flg22^417^, flg22^Pa^ or chitin. For treatment with live WCS417 cells, 7‐day‐old Col‐0 and *fls2* seedlings were transferred to new agar‐solidified 1 × MS plates with 0.5% sucrose. Two days later, roots were treated with WCS417 by applying 10 μl of a bacterial suspension (OD_600_ = 0.002) right below the root tip of each seedling. Seven days after the start of the treatments, shoot fresh weight, primary root length and number of lateral roots were determined essentially as described (Zamioudis *et al*., 2013).

### RNA sequencing

For RNA‐Seq analysis of root transcriptional profiles, roots of Col‐0 seedlings were collected in triplicate at 0, 0.5, 1, 3 and 6 h after treatment with live WCS417 cells or after treatment with the elicitors flg22^417^, flg22^Pa^ or chitin. Roots of untreated Col‐0 seedlings were collected at the same time points. Each of the three biological replicates per treatment and time point consisted of eight pooled root systems harvested from eight similarly‐treated plants. After harvest, root samples were snap‐frozen in liquid nitrogen and stored at −80°C.

Arabidopsis roots were homogenized using a mixer mill (Retsch) set to 30 Hz for 45 sec. RNA extraction, library preparation and RNA‐Seq were performed essentially as described (Coolen *et al*., 2016). Samples were sequenced by an Illumina NextSeq 500 platform using six sequencing runs. Within each run, samples were randomly assigned to seven lanes of the Illumina flow cells. In total, 12 randomized samples were loaded per lane of a NextSeq 500 V3 flow cell. To account for technical variation, each mix of 12 samples was sequenced in four different lanes over different flow cells. Three biological replicates of the five time points were sequenced in four technical replicates, resulting in ~30 million reads per sample with a read length of 75 bp single end. The raw RNA‐Seq read data are deposited in the Short Read Archive (http://www.ncbi.nlm.nih.gov/sra/; BioProject ID: PRJNA412447).

Processing of raw sequencing data, alignment of the RNA‐Seq data to the Arabidopsis genome and downstream processing were performed as described (Van Verk *et al*., 2013; Coolen *et al*., 2016; Hickman *et al*., 2017). RNA‐Seq reads were aligned to the TAIR version 10 version of the Arabidopsis genome using TopHat v2.0.4 (Trapnell *et al*., 2009) with parameters: ‘transcriptome‐mismatches 3’, ‘N 3’, ‘bowtie1’, ‘no‐novel‐juncs’, ‘genome‐read‐mismatches 3’, ‘read‐mismatches 3’, ‘G’, ‘min‐intron‐length 40’, ‘max‐intron‐length 2 000’. Gene expression levels were calculated by counting the number of mapped reads per annotated gene model using HTSeq‐count v0.5.3p9 (Anders *et al*., 2015). DESeq2 was used to calculated differential gene expression in the treatments relative to the respective control treatments (Love *et al*., 2014). For downstream analyses, raw read counts were normalized for between‐sample differences in sequencing depth (Love *et al*., 2014).

### GO analysis

Enriched GO‐terms in the different sets of DEGs were identified using ‘GO Term Finder’ (Boyle *et al*., 2004). This method tests for over‐representation of GO categories using the hypergeometric distribution and FDR for multiple testing (*P *≤ 0.05). Heatmaps of *P*‐values were generated using the R package (version 3.2.1).

### Analysis of DEG sets

Differentially expressed genes were processed with Virtual Plant web tool (http://virtualplant.bio.nyu.edu/cgi-bin/vpweb/virtualplant.cgi) in order to perform comparisons presented with Venn diagrams and identify overlapping gene sets between different treatments and treatment‐specific DEGs (Katari *et al*., 2010).

### Clustering of gene expression profiles

Clustering of DEGs was performed with SplineCluster (Heard *et al*., 2006) using log_2_‐fold change differences in expression between the live WCS417 or elicitor‐treated samples and their respective controls.

### qRT‐PCR analysis

Total RNA extraction for qRT‐PCR gene expression analysis was performed as described previously (Hickman *et al*., 2017). Transcript levels were calculated relative to the reference gene *At1g13320* (Czechowski *et al*., 2005) using the 2^−ΔCt^ method (Livak and Schmittgen, 2001; Schmittgen and Livak, 2008). The primers used for qRT‐PCR are provided in Table S1.

## Accession numbers

Arabidopsis gene names and identifiers referred to in the RNA‐Seq analysis are listed in Dataset S1 (Sheet ‘AGI’). Arabidopsis gene names and identifiers referred to in qRT‐PCR analyses are listed in Table S1.

## Author contributions

IAS, CZ and CMJP designed the research; IAS performed most of the research; IAS, SP, RH and MCvV performed data analysis; IAS, SP, RH, and CMJP wrote the manuscript. All authors discussed the results and commented on the manuscript.

## Supporting information


**Figure S1.** Alignment of flg22 peptides from flagellin of *P. simiae* WCS417 (flg22^417^) and *P. aeruginosa* PO1 (flg22^Pa^).
**Figure S2.** Venn diagrams of DEGs shared between Arabidopsis responses to flg22^Pa^ or chitin in different studies.

**Table S1.** List of primers used in this study.

**Dataset S1.** DEGs of *Arabidopsis thaliana* (AGI numbers of DEGs; FDR < 0.05; > twofold) in response to *P. simiae* WCS417, flg22^417^, flg22^Pa^ or chitin treatment at four consecutive time points.

**Dataset S2.** Lists of DEGs following flg22^Pa^ and chitin treatment from this study (after filtering out genes not present in microarray probesets) and from the studies of Zipfel *et al*. (2004) (flg22^Pa^), Beck *et al*. (2014) (flg22^Pa^) and Wan *et al*. (2008) (chitooctaose) that were used for the comparisons presented in Figure S2.

**Dataset S3.** Shared DEGs between WCS417, flg22^417^, flg22^Pa^ and chitin datasets shown in Figure 2b (shared DEGs between WCS417, flg22^417^, flg22^Pa^ and chitin treatments).

**Dataset S4.** Upregulated and downregulated DEGs only in response to flg22^417^ selected from the comparison with WCS417 (Figure 5) and the GO processes they are involved in.

**Dataset S5.** DEGs in roots 4 h after IAA treatment, DEGs from this study after root exposure to WCS417, their overlapping genes and the processes they are involved in.
