# Supplementary material for: Root transcriptional dynamics induced by beneficial rhizobacteria and microbial immune elicitors reveal signatures of adaptation to mutualists
Source: Plant J. 2017 Nov 15;93(1):166–80. doi: 10.1111/tpj.13741 (PMC5765484; doi:10.1111/tpj.13741)
Supplement: Supplementary file 1 — Figure S1. Alignment of flg22 peptides from flagellin of P. simiae WCS417 (flg22417) and P. aeruginosa PO1 (flg22Pa). Figure S2. Venn diagrams of DEGs shared between Arabidopsis responses to flg22Pa or chitin in different studies. [file TPJ-93-166-s001.pdf]

Supplemental Figures 1 and 2

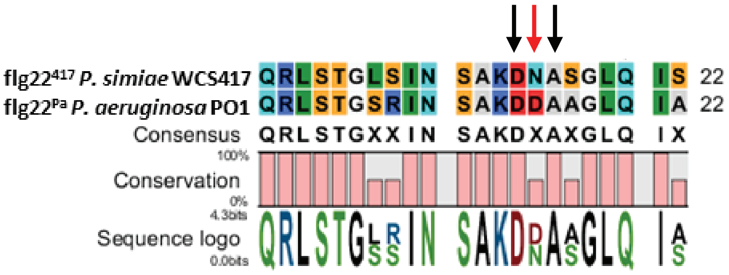

Supplemental Figure 1: **Alignment of flg22 peptides from flagellin of *P. simiae* WCS417 (flg22^{417}) and *P. aeruginosa* PO1 (flg22^{Pa}).** Arrows indicate the amino acids previously shown (Felix et al., 1999) to be required for the induction of immune responses in tomato. Alignment is created using the sequence alignment tool of CLC Main Workbench 6.9.

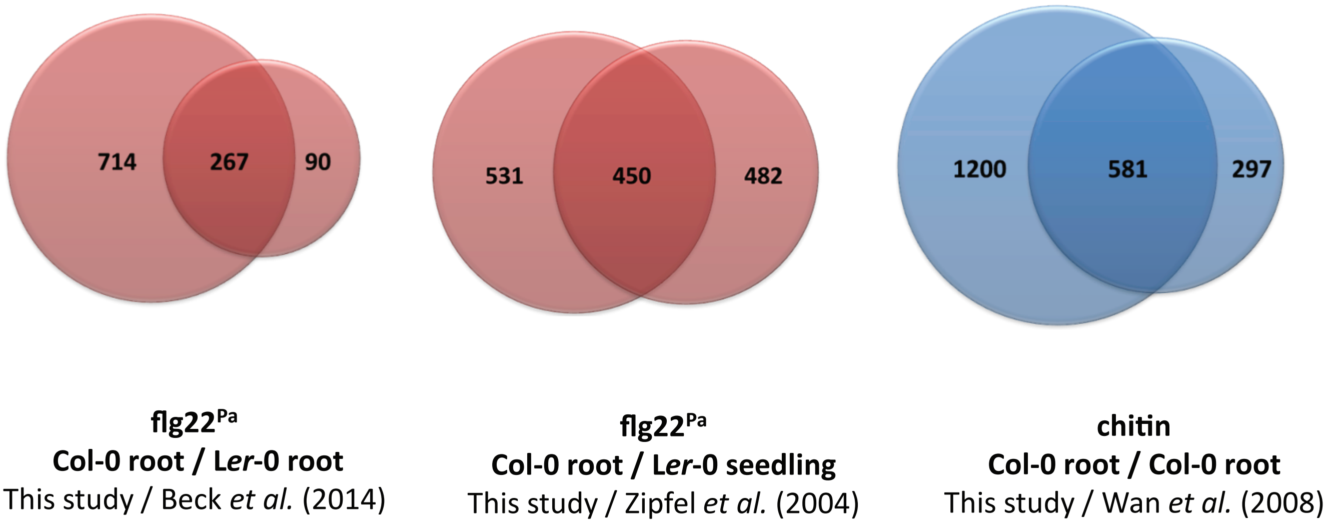

Supplemental Figure 2. Venn diagrams of DEGs shared between Arabidopsis responses to flg22^{Pa} or chitin in different studies. Venn diagrams show the overlap between DEGs responding to **(A)** flg22^{Pa}, 0.5 h after treatment of Col-0 roots (RNA-Seq data of this study) or L*er*-0 roots (Beck *et al.,* 2014), **(B)** flg22^{Pa}, 0.5 h after treatment of Col-0 roots (RNA-Seq data of this study) or L*er*-0 seedlings (Zipfel *et al.,* 2004), and **(C)** chitin, 0.5 h after treatment of Col-0 roots (RNA-Seq data of this study versus Wan *et al.,* 2008). In the study of Beck *et al.* (2014), L*er*-0 roots were treated with 10 μM flg22^{Pa}. In the study of Zipfel *et al.* (2004), whole L*er*-0 seedlings were treated with 10 μM flg22^{Pa}. In the study of Wan *et al.* (2008), Col-0 roots were treated with 1 μM chitooctaose. In all cases, DEGs were selected based on FDR <0.05 and $\log_2$-fold change >1.
