# Supplementary material for: Root transcriptional dynamics induced by beneficial rhizobacteria and microbial immune elicitors reveal signatures of adaptation to mutualists
Source: Plant J. 2017 Nov 15;93(1):166–80. doi: 10.1111/tpj.13741 (PMC5765484; doi:10.1111/tpj.13741)
Supplement: Supplementary file 2 — Table S1. List of primers used in this study. [file TPJ-93-166-s002.pdf]

Supplemental Table 1: List of primers used in this study

Primer name	Gene	Primer sequence
*PP2AA3*_Fw	At1g13320	5'-TAACGTGGCCAAAATGATGC-3'
*PP2AA3*_Rev	At1g13320	5'-GTTCTCCACAACCGCTTGGT-3'
*CYP71A12*_Fw	At2g30750	5'-GATTATCACCTCGGTTCCT-3'
*CYP71A12*_Rev	At2g30750	5'-CCACTAATACTTCCCAGATTA-3'
*MYB51*_Fw	At1g18570	5'-ACAAATGGTCTGCTATAGCT-3'
*MYB51*_Rev	At1g18570	5'-CTTGTGTGTAACTGGATCAA-3'
*SRO4*_Fw	At3g47720	5'-TTGGAGCACACACATGAACA-3'
*SRO4*_Rev	At3g47720	5'-CCAAGGAGATTTCGGGTTTT-3'
*SAD6*_Fw	At1g43800	5'-TCTTCTCAAACCCGTTGACC-3'
*SAD6*_Rev	At1g43800	5'-CTTTCCCTCAGCTCACGAAC-3'
*JAZ8*_Fw	At1g30135	5'-TGTGTTTTTCTTCAGATGTTACCC-3'
*JAZ8*_Rev	At1g30135	5'-TCTCTGCTTGCGATCGATATT-3'
*WRKY30*_Fw	At5g24110	5'-AGAGCGATGATTCCGATCAAG-3'
*WRKY30*_Rev	At5g24110	5'-CATCGTCCAGCGTTCTATCAA-3'
*MYB72*_Fw	At1g56160	5'-ACGAGATCAAAAACGTGTGGAAC-3'
*MYB72*_Rev	At1g56160	5'-TCATGATCTGCTTTTGTGCTTTG-3'
*IRT1*_Fw	At4g19690	5'-ACCCGTGCGTCAACAAAGCTAAAG-3'
*IRT1*_Rev	At4g19690	5'-TCCCGGAGGCGAAACACTTAATGA-3'
*GH3.3*_Fw	At2g23170	5'-CATCACAGAGTTCCTCACAAGC-3'
*GH3.3*_Rev	At2g23170	5'-GTCGGTCCATGTCTTCATCA-3'
