# Supplementary material for: Root transcriptional dynamics induced by beneficial rhizobacteria and microbial immune elicitors reveal signatures of adaptation to mutualists
Source: Plant J. 2017 Nov 15;93(1):166–80. doi: 10.1111/tpj.13741 (PMC5765484; doi:10.1111/tpj.13741)
Supplement: Supplementary file 8 [file TPJ-93-166-s008.docx]

**supporting information legends**

**Supplemental Figure 1.** Alignment of flg22 peptides from flagellin of *P. simiae* WCS417 (flg22^417^) and *P. aeruginosa* PO1 (flg22^Pa^). Arrows indicate the amino acids previously shown

(Felix *et al*., 1999) to be required for the induction of immune responses in tomato. Alignment is created using the sequence alignment tool of CLC Main Workbench 6.9.

**Supplemental Figure 2.** Venn diagrams of DEGs shared between Arabidopsis responses to flg22^Pa^ or chitin in different studies. Venn diagrams show the overlap between DEGs responding to **(A)** flg22Pa, 0.5 h after treatment of Col-0 roots (RNA-Seq data of this study) or L*er*-0 roots (Beck *et al.,* 2014), **(B)** flg22Pa, 0.5 h after treatment of Col-0 roots (RNA-Seq data of this study) or L*er*-0 seedlings (Zipfel *et al.,* 2004), and **(C)** chitin, 0.5 h after treatment of Col-0 roots (RNA-Seq data of this study versus Wan *et al.,* 2008). In the study of Beck *et al.* (2014), L*er*-0 roots were treated with 10 μM flg22Pa. In the study of Zipfel *et al.* (2004), whole L*er*-0 seedlings were treated with 10 μM flg22Pa. In the study of Wan *et al.* (2008), Col-0 roots were treated with 1 μM chitooctaose. In all cases, DEGs were selected based on FDR <0.05 and log2-fold change >1.

**Supplemental Table 1.** List of primers used in this study.

**Supplemental Dataset 1.** Differentially expressed genes (DEGs) of Arabidopsis thaliana (AGI numbers of DEGs; FDR <0.05; >2-fold) in response to *P. simiae* WCS417, flg22^417^, flg22^Pa^, or chitin treatment at four consecutive time points.

**Supplemental Dataset 2.** Lists of DEGs following flg22^Pa^ and chitin treatment from this study (after filtering out genes not present in microarray probesets) and from the studies of Zipfel et al., 2004 (flg22^Pa^), Beck et al., 2014 (flg22^Pa^) and Wan et al. 2008 (chitooctaose) that were used for the comparisons presented in Supplemental Figure 2.

**Supplemental Dataset 3.** Shared DEGs between WCS417, flg22^417^, flg22^Pa^ and chitin datasets shown in Figure 2B (Shared DEGs between WCS417, flg22^417^, flg22^Pa^ and chitin treatments).

**Supplemental Dataset 4.** Up-regulated and down-regulated DEGs only in response to flg22^417^ selected from the comparison with WCS417 (Figure 5) and the GO processes they are involved in.

**Supplemental Dataset 5.** DEGs in roots 4 h after IAA treatment, DEGs from this study after root exposure to WCS417, their overlapping genes and the processes they are involved.
